# Photosensitizer-Embedded Polyacrylonitrile Nanofibers as Antimicrobial Non-Woven Textile

**DOI:** 10.3390/nano6040077

**Published:** 2016-04-20

**Authors:** Sarah L. Stanley, Frank Scholle, Jiadeng Zhu, Yao Lu, Xiangwu Zhang, Xingci Situ, Reza A. Ghiladi

**Affiliations:** 1 Department of Chemistry, North Carolina State University, Raleigh, NC 27695-8204, USA; slstanl2@ncsu.edu (S.L.S.); xsitu1@gmail.com (X.S.); 2 Department of Biological Sciences, North Carolina State University, Raleigh, NC 27695-7614, USA; fscholl@ncsu.edu; 3 Fiber and Polymer Science Program, Department of Textile Engineering, Chemistry and Science, North Carolina State University, Raleigh, NC 27695-8301, USA; jzhu14@ncsu.edu (J.Z.); ylu14@ncsu.edu (Y.L.); xzhang13@ncsu.edu (X.Z.)

**Keywords:** photodynamic inactivation, singlet oxygen, antibacterial, antiviral, photobiocidal, polyacrylonitrile, photosensitizer, porphyrin

## Abstract

Toward the objective of developing platform technologies for anti-infective materials based upon photodynamic inactivation, we employed electrospinning to prepare a non-woven textile comprised of polyacrylonitrile nanofibers embedded with a porphyrin-based cationic photosensitizer; termed PAN-Por^(+)^. Photosensitizer loading was determined to be 34.8 nmol/mg material; with thermostability to 300 °C. Antibacterial efficacy was evaluated against four bacteria belonging to the ESKAPE family of pathogens (*Staphylococcus aureus*; vancomycin-resistant *Enterococcus faecium*; *Acinetobacter baumannii*; and *Klebsiella pneumonia*), as well as *Escherichia coli*. Our results demonstrated broad photodynamic inactivation of all bacterial strains studied upon illumination (30 min; 65 ± 5 mW/cm^2^; 400–700 nm) by a minimum of 99.9996+% (5.8 log units) regardless of taxonomic classification. PAN-Por^(+)^ also inactivated human adenovirus-5 (~99.8% reduction in PFU/mL) and vesicular stomatitis virus (>7 log units reduction in PFU/mL). When compared to cellulose-based materials employing this same photosensitizer; the higher levels of photodynamic inactivation achieved here with PAN-Por^(+)^ are likely due to the combined effects of higher photosensitizer loading and a greater surface area imparted by the use of nanofibers. These results demonstrate the potential of photosensitizer-embedded polyacrylonitrile nanofibers to serve as scalable scaffolds for anti-infective or self-sterilizing materials against both bacteria and viruses when employing a photodynamic inactivation mode of action.

## 1. Introduction

According to the center for disease control’s (CDC’s) Healthcare-Associated Infections (HAI) Prevalence Survey, there were an estimated 722,000 HAIs in U.S. acute care hospitals in 2011, equivalent to ~1 out of every 25 inpatients having at least one health care–associated infection on any given day [1]. Approximately 75,000 deaths were attributed to these infections, or about 10% of the total HAIs. When one considers that these estimates of the national burden of health care–associated infections were limited to acute care hospitals, factoring in the magnitude of HAIs attributed to other settings (e.g., skilled nursing facilities, outpatient clinics, urgent care facilities) further highlights the scope and staggering cost of nosocomial infections. One of the main contributing factors to HAIs is the ability of pathogens such as bacteria, fungi, and viruses to adhere to, and survive on, surfaces that leads to their subsequent transmission to new hosts [2,3,4,5]. As an example, *Staphylococcus aureus* can survive for weeks to months under dry conditions on the cotton and polyester fabrics used in hospitals [6,7]. Though typically not a concern for healthy individuals, a second factor that contributes to HAIs is drug resistance, and five classes of antibiotic-resistant pathogens in particular have emerged as major public health threats: vancomycin-resistant enterococci (VRE), methicillin-resistant *Staphylococcus aureus* (MRSA), multidrug-resistant mycobacteria, Gram-negative bacteria, and fungi [8]. To combat these contributing factors to HAIs, more research into effective surface disinfection and alternative materials (fabrics, plastics or coatings) with antimicrobial properties capable of overcoming drug-resistance is needed. Moreover, food processing, packaging and service industries, waste water treatment, daycare facilities, and personal households are other areas where infectious agents are easily spread, but may be countered by an anti-infective coating.

In general terms, an ideal anti-infective surface necessitates a permanent or rechargeable anti-infective character, is harmless to the environment, operates in a user-independent manner, and effectively leads to the eradication of a broad scope of pathogens [9,10,11]. Several classes of antimicrobial agents are currently being investigated or are commercially available [12,13,14,15,16,17,18,19,20,21], yet have disadvantages such as the loss of antimicrobial activity by leaching of the biocide, consumption of the germicidal ability, environmentally hazardous agents, dependency on direct contact of the antimicrobial entity with the microorganism, and/or their efficacy is often limited to a single class of microbe (*i.e.*, only bacteria or fungi). A promising area that circumvents many of these shortcomings while at the same time satisfying many of the aforementioned criteria of an ideal anti-infective material is antimicrobial photodynamic inactivation (aPDI) [22,23,24,25,26]. aPDI employs light, air, and a photosensitizer (PS) to generate primarily singlet oxygen (^1^O_2_) as the biocidal agent, and represents a complementary strategy for the treatment of microbial infections [27,28]. Advantages of materials-based aPDI include (i) employing singlet oxygen as the biocidal agent (which, given its short lifetime and decay to harmless oxygen as an end product [29] can be considered environmentally benign); (ii) multiple routes to PS incorporation, including the attachment of the PS through electrostatic interactions [30], encapsulation within a polymeric matrix [30,31,32,33,34,35,36], or direct attachment via a covalent bond (prevents leaching into the environment) [37,38,39,40,41,42,43,44]; (iii) the ability of the PS to potentially function in the absence of direct contact with the pathogen due to the diffusibility of singlet oxygen [40,45]; and (iv) of great importance with respect to nosocomial infections is that singlet oxygen or other photo-generated reactive oxygen species cause non-specific damage from which microbial resistance is unlikely to arise [27,46,47]. To this latter point, aPDI has been shown to possess broad-spectrum antibacterial [23,27,48,49,50], antiviral [31,51,52,53], antifungal [46,54,55,56], and antiparasitic [57,58] properties. Finally, as aPDI employs harmless white light [10], it has the advantage over ultraviolet-C (UVC) (as an example of another light-based sterilization technique), vaporized hydrogen peroxide, or chlorine dioxide in that it can function without the need for protecting people against the deleterious effects of the biocidal agent.

A number of materials based upon a photodynamic mode of action have been recently reported. These include: synthetic polymer materials (polyurethane, polystyrene, polycaprolactone and polyamide-6) with encapsulated photosensitizers (e.g., free-base or zinc tetraphenylporphyrin, zinc phthalocyanine, cationic 5,10,15,20-tetrakis(1-methylpyridinium-4-yl)porphyrin (TMPyP)) that exhibit photobactericidal (*E. coli*) and photovirucidal (against non-enveloped polyomavirus and enveloped baculovirus) efficacy [30,31,32,33,34,35,36], as well as natural polymer materials based on cellulose nanocrystals (Por^(+)^-CNCs [37,38,59]), cellulose fibers (Por^(+)^-paper [39]), or cotton fabrics [42,44] that possess broad anti-infective efficacy against both bacteria (e.g., *Staphylococcus aureus*, vancomycin-resistant *Enterococcus faecium*, *Acinetobacter baumannii*, *Pseudomonas aeruginosa*, and *Klebsiella pneumonia*) and viruses (e.g., dengue-1, influenza A, and human adenovirus-5). However, further studies regarding the design, scope, and potential applications of these materials remain. These include: (i) better defining the scope of antibacterial activity for synthetic nanofiber materials as their photobactericidal studies were primarily limited to *E. coli*, (ii) direct comparison of synthetic *vs.* natural polymers as scaffolds for photoactive materials, and (iii) gaining additional insight into whether more facile photosensitizer embedding/encapsulation strategies are sufficient for material design, or are covalent approaches necessary for maximum anti-infective efficacy.

To begin to address these areas systematically, the objective of the present study was to prepare polyacrylonitrile nanofibers embedded with a photosensitizer via electrospinning, and to study the anti-infective properties of the resulting non-woven textile, termed PAN-Por^(+)^, against both Gram-positive and Gram-negative bacteria of the ESKAPE family of pathogens [60], as well as to extend these photoinactivation studies against viruses (ESKAPE = acronym for the most common nosocomial infectious agents, *Enterococcifaecium*, *Staphylococcusaureus*, *Klebsiellapneumoniae*, *Acinetobacterbaumannii*, *Pseudomonasaeruginosa*, and *Enterobacter* species). The strategy employed enables the comparison of the antimicrobial efficacy of PAN-Por^(+)^ to similar cellulose-based counterparts (Por^(+)^-CNCs [37,38] and Por^(+)^-paper [39]) that our lab has recently studied. As we are employing the same porphyrin-based cationic photosensitizer (*i.e.*, Por^(+)^), such a comparison will enable us to better understand how the use of synthetic (polyacrylonitrile) nanofibers *vs.* natural (cellulose) fibers impacts the design of antimicrobial materials, as well as enable a direct comparison of the more facile embedded photosensitizer approach used in the preparation of PAN-Por^(+)^ against the more synthetically challenging covalent attachment method used in Por^(+)^-CNCs and Por^(+)^-paper.

## 2. Results and Discussion

### 2.1. Electrospinning and Characterization of PAN-Por^(+)^ Nanofibers

#### 2.1.1. Scanning Electron Microscopy (SEM) of PAN-Por^(+)^

**Figure 1:**
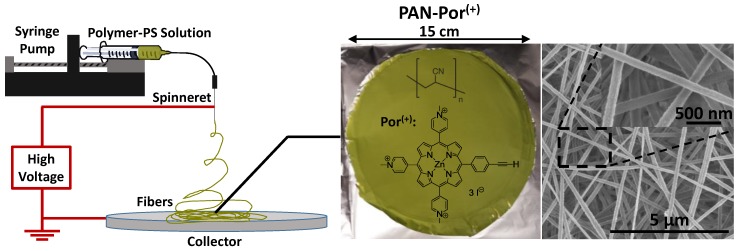
Electrospinning schematic (**left**), PAN-Por^(+)^ (**middle**), and scanning electron microscopy (SEM) images (**right**).

The preparation of PAN-Por^(+)^ nanofibers was accomplished by thoroughly mixing precursor Por^(+)^ and PAN prior to electrospinning. Application of a high voltage and collection on an aluminum foil target yielded the green PAN-Por^(+)^ nanofiber material as shown in Figure 1. Characterization by scanning electron microscopy (SEM) of both the PAN-Por^(+)^ nanofibers as well as the photosensitizer-free (control) PAN nanofibers (Figure S1) revealed that both materials were highly similar overall, consisting of randomly arranged fibers with an average diameter of ~175 nm. The high-resolution SEM images (Figure 1 and Figure S1, inserts) demonstrate that the nanofibers of PAN-Por^(+)^ and PAN have surfaces of equivalent smoothness, suggesting that presence of the cationic Por^(+)^ photosensitizer does not impact the morphology of the resulting nanofibers.

#### 2.1.2. Determination of Photosensitizer Loading in PAN-Por^(+)^

Prior to the determination of photosensitizer loading and subsequent antimicrobial studies, each sample of PAN-Por^(+)^ was washed, thereby removing adventitiously bound Por^(+)^ to a concentration of less than 19 nM as determined by UV-visible spectroscopy (*vide infra*). To determine the extent of porphyrin loading, washed PAN-Por^(+)^ was dissolved in dimethylformamide (DMF), solubilizing both the PAN nanofibers and the Por^(+)^ photosensitizer, thereby enabling the photosensitizer content to be determined by UV-visible spectroscopy to be 34.8 ± 0.1 nmol Por^(+)^/mg PAN-Por^(+)^, approximately three-fold higher than Por^(+)^-paper (12.4 nmol Por^(+)^/mg material [39]), but less than the 160 nmol Por^(+)^/mg material in Por^(+)^-CNCs [37]. The value of 34.8 ± 0.1 nmol Por^(+)^/mg PAN-Por^(+)^ represents 3.9% of the overall mass of PAN-Por^(+)^ nanofibers (based on the triiodide salt of Por^(+)^ [37,61]), and suggests that of the 10 wt % with respect to the mass of PAN that was used in the electrospinning of PAN-Por^(+)^, that 6.1% of the Por^(+)^ was adventitiously bound and removed during the washing procedure.

#### 2.1.3. Thermal Gravimetric Analysis (TGA) of PAN-Por^(+)^

Thermal gravimetric analysis was performed on the unmodified PAN mother fibers and PAN-Por^(+)^ to gain an understanding of the thermal stability of these materials (Figure S2). Overall, both unmodified PAN and PAN-Por^(+)^ nanofibers exhibited virtually identical thermostability, with negligible weight losses of 1.1% and 0.5%, respectively, up to 300 °C, a result that was not wholly unexpected: while the Por^(+)^ photosensitizer has been shown to lose 30% of its mass between 180 and 300 °C (and ultimately *ca*. 42.5% loss at 330 °C [37]), the level of Por^(+)^ photosensitizer loading (34.8 nmol Por^(+)^/mg PAN-Por^(+)^ nanofibers, *vide supra*) represents only 3.9% of the overall mass of PAN-Por^(+)^ nanofibers. As such, a 1.2% overall mass loss (at most) was expected based on the known behavior of Por^(+)^, compared with the 0.5% loss observed, the difference of which is likely due to the error inherent in TGA measurements, although we cannot conclusively rule out that the nanofibers themselves inhibit thermal decomposition of Por^(+)^. By way of comparison to the cellulose based analogs, Por^(+)^-paper displayed a similar thermostability (no significant mass reduction was observed until 290 °C [39]), whereas Por^(+)^-CNCs afforded a material with a much greater weight loss of ~20% observed at 300 °C [37].

### 2.2. Antibacterial Photodynamic Inactivation Studies

#### 2.2.1. *In vitro* aPDI Studies against Gram-Positive and Gram-Negative Bacteria

**Figure 2:**
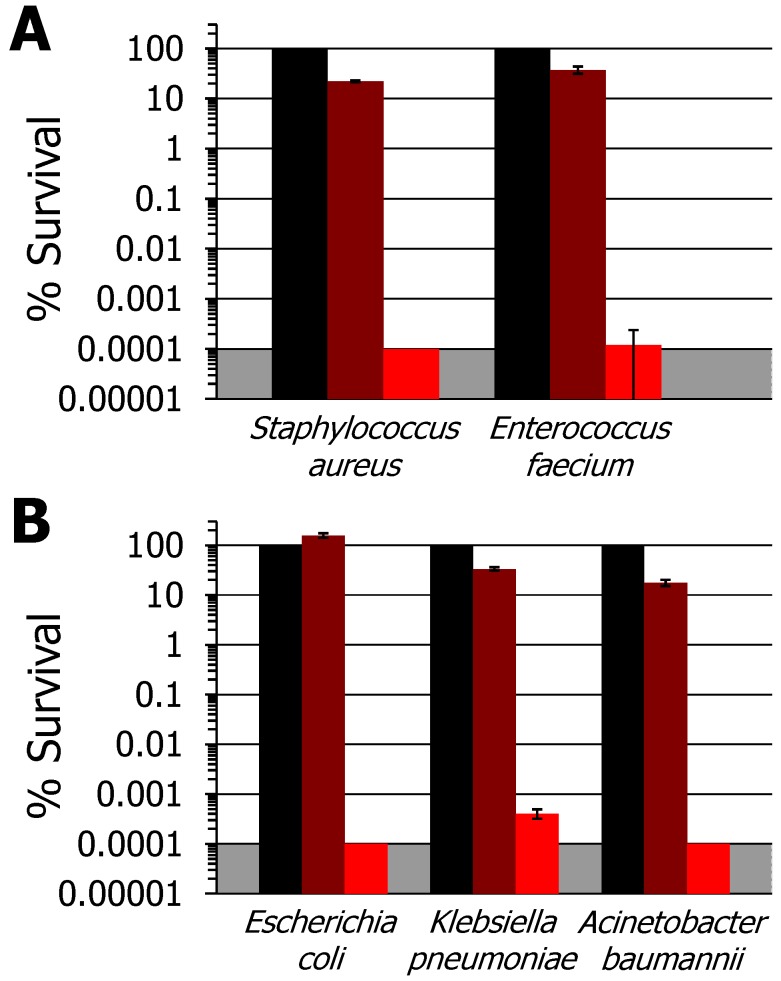
Photodynamic inactivation studies employing PAN-Por^(+)^. (**A**) Gram-positive species: methicillin-susceptible *S. aureus* (MSSA) ATCC-2913 and the vancomycin-resistant *E. faecium* (VRE) ATCC-2320 strain. (**B**) Gram-negative species: *E. coli* BL21-(Dε3)pLysS, *K. pneumoniae* ATCC-2146, and *A. baumannii* ATCC-19606. For both panels, displayed are the material-free (cells-only) dark control set to 100% (black), as well as the dark control of PAN-Por^(+)^ (maroon) and the illuminated PAN-Por^(+)^ (red) studies, both as the percent survival of the material-free (cells-only) dark control. For all bacteria, the illumination conditions were as follows: 30 min, 400–700 nm, 65 ± 5 mW/cm^2^ (total fluence of 118 J/cm^2^). As the plating technique employed to determine % survival did not allow for detection of survival rates of <0.0001%, data points below the detection limit were set to 0.0001% survival for graphing purposes and are indicated by the grey shaded area. In the cases where error bars cannot be visualized, the error bars themselves were smaller than the marker employed in the plot.

*In vitro* aPDI studies employing PAN-Por^(+)^ were performed under fixed illumination conditions (30 min, 400–700 nm, 65 ± 5 mW/cm^2^) to enable comparisons with previous studies that employed the identical photosensitizer covalently appended to cellulose nanocrystals (as Por^(+)^-CNCs [37,38]) and cellulose fibers (as Por^(+)^-paper [39]). The two Gram-positive bacteria, *S. aureus* ATCC-2913 and the vancomycin-resistant *E. faecium* (VRE) strain ATCC-2320, were found to be highly susceptible to photodynamic inactivation with PAN-Por^(+)^: upon illumination, *S. aureus* was fully inactivated to the detection limit (>99.9999+%, 6 log units, *p* < 0.005; Figure 2A, red), and vancomycin-resistant *E. faecium* (ATCC-2320) was inactivated by 99.9998% (~5.9 log units, *p* < 0.005; Figure 2A, red). By contrast, all bacteria exhibited 100% survival on the unmodified PAN (PS-free control) under the same illumination conditions (see Figure S3 for representative data against *K. pneumonia*), demonstrating the requirement of the photosensitizer for bacteria inactivation. Although <1 log unit, the dark controls employing PAN-Por^(+)^ also exhibited inactivation (Figure 2, maroon), likely due to the photodynamic production of singlet oxygen by the material even when minimizing the ambient room light during the assay, although we cannot conclusively rule out a slight dark toxicity of the material. Photodynamic inactivation under dark control conditions has been noted previously for Por^(+)^-paper [39], but could be circumvented in that study by using a darkroom red light that emitted at wavelengths where the photosensitizer did not absorb well. Here, we suggest that the ~3× higher loading of the PS in PAN-Por^(+)^ when compared with Por^(+)^-paper leads to this material having a very high sensitivity to even such minimal light conditions employed during the serial dilution and other workup steps of the assay. By way of comparison for *S. aureus*, PAN-Por^(+)^ achieved a comparable level of inactivation as Por^(+)^-CNC (~6 log units, [37,38]), and was superior to Por^(+)^-paper (~5 log units, [39]). Moreover, while not previously tested against Por^(+)^-CNCs, against the *E. faecium* strain PAN-Por^(+)^ also achieved a far higher level of inactivation than Por^(+)^-paper (4 log unit CFU reduction, [39]).

When explored against Gram-negative bacteria, PAN-Por^(+)^ showed virtually identical inactivation efficacy (Figure 2B) when compared to the Gram-positive strains above under the same illumination conditions. Generally speaking, this was unexpected as the additional outer membrane of highly impermeable lipopolysaccharides present in Gram-negative bacteria makes them more resistant to photodynamic inactivation than Gram-positive species [62], but we have noted this previously for Por^(+)^-paper (but not for Por^(+)^-CNCs) [39]. Specifically, we observed here that both *Escherichia coli* BL21-(Dε3)pLysS and *Acinetobacter baumannii* ATCC-19606 were fully inactivated to the detection limit (>99.9999+%, 6 log units, *p* < 0.005; Figure 2B, red), and the multi-drug resistant NDM-1-producing *K. pneumoniae* clinical isolate ATCC-2146 was inactivated to 99.9996+% reduction (~5.8 log units; *p* < 0.005). By way of comparison, PAN-Por^(+)^ was able to achieve a modestly improved level of inactivation for *Acinetobacter baumannii* ATCC-19606 than either Por^(+)^-paper (~5 log units [39]) or Por^(+)^-CNCs (5.5 log units [38]), and was significantly superior against the *K. pneumoniae* clinical isolate ATCC-2146 than Por^(+)^-paper (4.5 log units [39]). Even more impressively, PAN-Por^(+)^ was far more effective against *E. coli* than Por^(+)^-CNCs (1.8 log units reduction [37]). Note that neither *K. pneumoniae* clinical isolate ATCC-2146 nor *E. coli* were studied with Por^(+)^-CNCs or Por^(+)^-paper, respectively, thereby preventing these comparisons.

Finally, the effects of extended photobleaching were examined by pre-illuminating PAN-Por^(+)^ continuously for 8 h under identical illumination conditions (400–700 nm, 65 ± 5 mW/cm^2^), followed by repetition of the *K. pneumoniae* trial employing this pre-illuminated (“photo-aged”) paper. Inactivation to 99.998% (~5 log units, *p* < 0.005) was observed (Figure S4), suggesting the material has excellent robustness with respect to photobleaching over the time period examined.

Taken together, the results demonstrate that PAN-Por^(+)^ was able to achieve a comparable or superior level of photodynamic inactivation against the Gram-positive and Gram-negative bacteria examined here when compared with Por^(+)^-paper or Por^(+)^-CNCs, while at the same time has the advantage in that it avoids the necessity of the tedious covalent attachment of the photosensitizer to the polymeric cellulose scaffold employed in those other materials. Based on the results, we surmise that the higher efficacy of PAN-Por^(+)^ is likely due to two factors: (i) the ~3× higher loading of the Por^(+)^ PS in PAN-Por^(+)^ when compared with Por^(+)^-paper likely leads to a higher production of bactericidal singlet oxygen (or other ROS), and (ii) the use of nanofibers in PAN-Por^(+)^ generates a material with a greater surface area than the 10–50 µm diameter cellulose fibers [63] that are typical for materials such as Por^(+)^-paper. Overall, our results against both Gram-positive and Gram-negative bacteria are particularly noteworthy in the context of ‘ESKAPE’ pathogens [39,60] given the broad, detection-limit photoinactivation efficacy of PAN-Por^(+)^ against the bacterial strains examined here regardless of their taxonomic classification.

#### 2.2.2. Solution Studies with Por^(+)^

**Figure 3:**
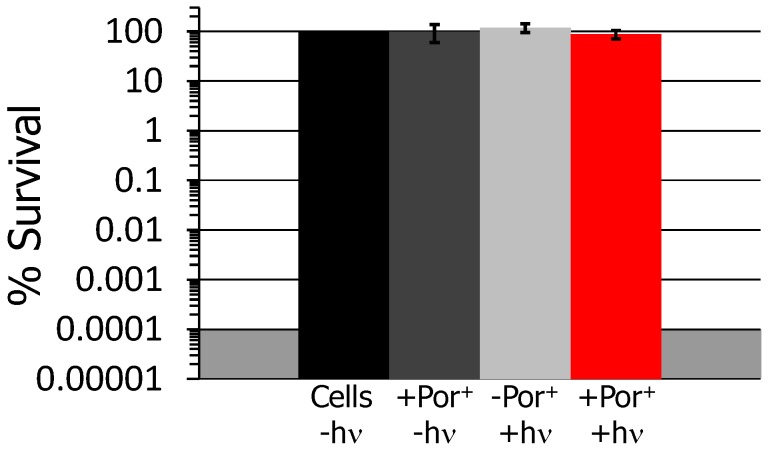
Photodynamic inactivation of *Klebsiella pneumoniae* using 50 nM Por^(+)^ demonstrating that this photosensitizer (PS) concentration was unable to photoinactivate the bacterium. Displayed are the dark PS-free (cells-only) control set to 100% (black), the % survival of the dark control of Por^(+)^ as a percent of the dark PS-free control (dark grey), the illuminated PS-free control as a percent of the dark PS-free control (light grey), and the illuminated Por^(+)^ as a percent of the dark PS-free control (red). The illumination conditions and error bar visualizations were as described in Figure 2.

UV-visible spectroscopic analysis of the washed PAN-Por^(+)^ revealed that the washing procedure removed adventitiously bound Por^(+)^ to a concentration of less than 19 nM. Although minor, the question arises as to whether even such a low level (≤19 nM) of adventitiously bound photosensitizer would photoinactivate bacteria. Thus, a solution study was performed with 50 nM Por^(+)^, a higher concentration than present in the washed material, using the multi-drug resistant NDM-1-producing *K. pneumoniae* clinical isolate ATCC-2146 as our model organism under fixed illumination conditions (30 min, 400–700 nm, 65 ± 5 mW/cm^2^). Gratifyingly, with 100% cell survival noted for all conditions studied, no statistically significant inactivation of *K. pneumoniae* was observed (Figure 3), and the results of the solution study confirm that the results of our materials-based antimicrobial studies (*vide supra*) were due to embedded photosensitizer only, and not a solution-derived result from adventitiously bound photosensitizer at ≤19 nM. Additionally, it is important to note that solution studies with the structurally-related benchmark photosensitizer TMPyP [meso-tetrakis(1-methylpyridinium-4-yl)porphyrin] have shown no significant (*i.e.*, <0.5 log unit inactivation) photoinactivation of any of the bacteria studied here at 100 nM photosensitizer concentration (specifically Figure S4 in reference [50] and Figure 2.4A in reference [59]).

### 2.3. Antiviral Photodynamic Inactivation Studies

**Figure 4:**
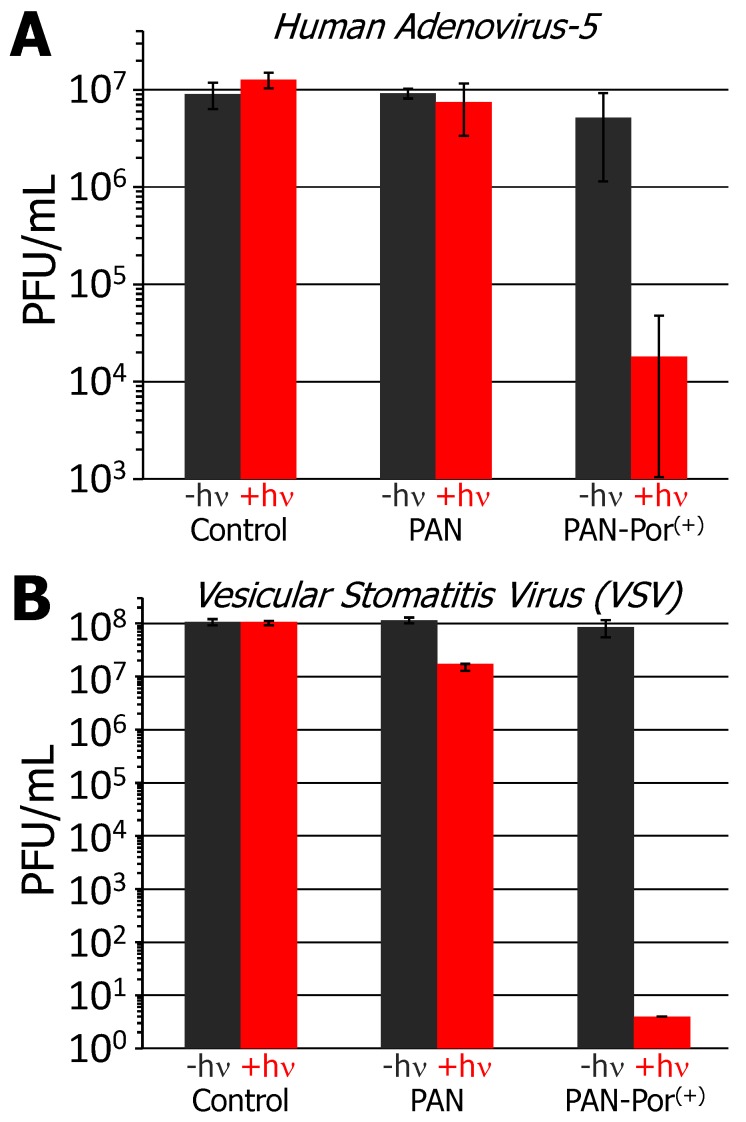
Antiviral photodynamic inactivation studies employing PAN-Por^(+)^ against (**A**) human adenovirus-5 (HAd-5) and (**B**) vesicular stomatitis virus (VSV). The black and red bars represent the number of PFU/mL of the non-illuminated (dark) and illuminated conditions, respectively, for the material-free (control), photosensitizer-free (PAN only) control, and PAN-Por^(+)^ studies. The illumination conditions and error bar visualizations were as described in Figure 2.

PAN-Por^(+)^ was examined for its antiviral PDI activity against two viruses: the non-enveloped human adenovirus-5 (HAd-5; Figure 4A) and enveloped vesicular stomatitis virus (VSV; Figure 4B). No inactivation of either virus was seen in the absence of material (material-free control) for either illuminated or non-illuminated conditions. In the presence of electrospun polyacrylonitrile nanofibers (PS-free PAN control), no statistically significant inactivation of either virus was observed under non-illuminated conditions, nor was any observed for HAd-5 upon illumination; only a minor <1 log unit reduction in PFU/mL was observed for VSV in the presence of illuminated PAN. Notably, however, upon illumination (30 min, 400–700 nm, 65 ± 5 mW/cm^2^) in the presence of PAN-Por^(+)^, human adenovirus-5 was inactivated to ~99.8% reduction in PFU/mL (~2.9 log units; *p* < 0.01, Figure 4A), and represents approximately an order of magnitude improvement when compared to the results obtained for Por^(+)^-paper (~99% reduction in FFU/mL, 2 log units [39]). Even more impressive were the results obtained for vesicular stomatitis virus: inactivation of the virus exceeding >7 log units reduction in PFU/mL was noted upon illumination for PAN-Por^(+)^ (Figure 4B). We surmise that the difference in inactivation efficacy between these two viruses is likely due to the nature of the virus itself with respect to the viral envelope: the protein-based capsids of non-enveloped viruses may be more resistant to photosensitization than the lipid-bilayer-enclosed enveloped viruses, an observation that we noted previously in our studies with Por^(+)^-paper [39], and that others have noted as well [51,64,65]. As was noted for the antibacterial studies, no statistically significant reduction for either virus was noted in the presence of PAN-Por^(+)^ under non-illuminated conditions, again demonstrating the requirement of the photosensitizer for significant virus inactivation. Finally, as was also noted for the antibacterial studies, the improved efficacy of PAN-Por^(+)^ when compared to Por^(+)^-paper is likely due to the combined effects of higher photosensitizer loading and a potentially greater surface area due to the use of nanofibers (PAN) over the larger cellulose fibers.

## 3. Experimental Section

### 3.1. Materials and Methods

Buffer salts were purchased from Fisher Scientific (Pittsburgh, PA, USA), Nutrient Broth #234000 was obtained from BD Difco (Franklin Lakes, NJ, USA), LB broth Miller from EMD Chemicals (Billerica, MA, USA), and Tryptic Soy Broth from Teknova (Hollister, CA, USA). Unless otherwise specified, all other chemicals were obtained from commercial sources in reagent grade purity or better. Deionized water was used for all media and buffers. UV-visible absorption measurements were performed on a Varian Cary 50 Bio instrument or a Genesys 10 UV scanning spectrophotometer from Thermo Electron Corp (Waltham, MA, USA) for single wavelength measurements. The photosensitizer Por^(+)^ was synthesized as described previously [37,38,59]. Field-emission scanning electron microscopy (FE-SEM, FEI Verios 460L, Hillsboro, OR, USA) was performed at an acceleration voltage of 2 kV to observe the morphology of the obtained materials. Thermal gravimetric analysis (TGA) was carried out on a TA instrument TGAQ50 (New Castle, DE, USA) ramping 8 °C/min under N_2_ purging.

### 3.2. Electrospinning of PAN-Por^(+)^

Polyacrylonitrile (PAN, M_w_ = 150,000) and *N*,*N*-dimethylformamide (DMF) were purchased from Sigma-Aldrich (St. Louis, MO, USA) and used as received. The solution was prepared by firstly dissolving PAN powder into DMF solvent with a weight percentage of 5 wt %. The PAN/DMF solution was stirred for over 24 h, followed by the addition of cationic porphyrin (1130 g/mol). The mass of cationic porphyrin was 10 wt % with respect to the mass of PAN. The solution was further stirred for 24 h prior to electrospinning in an apparatus that employed a Gamma ES40P-20 W/DAM variable high voltage (15 kV) power supply. The flow rate applied was 0.75 mL/h. The needle-to-collector distance was set at 15 cm and electrospun fibers were collected on an aluminum foil. Prior to characterization and aPDI testing, the material was subjected to the following washing procedure: a sample of PAN-Por^(+)^ (~1 mg) was placed in a 24-well plate, and a minimum of 2 mL deionized water was added by pipet. Agitation (swirling by hand or by pipet) proceeded for 30 s, followed by removal of the solution. These steps were repeated for a total of eight iterations. Using this procedure, adventitiously bound Por^(+)^ was decreased to a concentration of less than 19 nM as determined by UV-visible spectroscopy.

### 3.3. Determination of Porphyrin Loading

Approximately 9 mg of dry, washed textile was dissolved in 4 mL dimethylformamide and 6 mL deionized water, thereby fully solubilizing the Por^(+)^ photosensitizer. The resulting solution was syringe filtered (0.22 µm) to remove any trace of undissolved PAN nanofibers, diluted 1:6 with deionized water, and the concentration of the Por^(+)^ was determined by UV-visible spectroscopy using ε_Soret_ = 195,000 M^−1^·cm^−1^ [38,59].

### 3.4. Cell culture

Bacteria were cultured at 37 °C in 5 mL culture tubes on an orbital shaker at 500 RPM. The following growth conditions were employed: methicillin susceptible *Staphylococcus aureus* 2913 was cultured without antibiotics in tryptic soy broth; vancomycin resistant *Enterococcus faecium* (ATCC-2320) was cultured with 50 µg/mL ampicillin in DB Difco Bacto Brain Heart Infusion 237500; *Escherichia coli* BL21-(Dε3)pLysS (Stratagene, San Diego, CA, USA) was grown in Miller LB media with 100 µg/mL ampicillin; *Acinetobacter baumannii* (ATCC-19606) was grown in Miller LB media without antibiotics. *Klebsiella pneumoniae* (ATCC-2146) was cultured with 100 µg/mL ampicillin in DB Difco Nutrient broth #234000. A Genesys 10 UV scanning spectrophotometer was employed to spectrophotometrically follow the growth of each bacterium to a concentration of 1–4 × 10^8^ CFU/mL, after which the bacteria were pelleted (15 min, ~4150 g) by centrifugation, and the supernatant was removed via decantation. The bacteria were then resuspended in broth media (5 mL) and diluted to ~10^8^ CFU/mL (determined spectrophotometrically).

### 3.5. Viral Propagation

Vero cells were employed to propagate (and titer by plaque assay) vesicular stomatitis virus (VSV) NJ strain. The human lung carcinoma cell line A549 was used to propagate and titer human adenovirus-5 (HAd-5). Plaques were visualized by crystal violet staining.

### 3.6. Photodynamic Inactivation Assay

A LumaCare USA model LC122 PDT non-coherent light source was employed for all antimicrobial photodynamic inactivation studies. The lamp was equipped with an OSRAM 64653 HLX Xenophot bulb (250 W, 24 V), and employed a LUM V (400–700 nm band pass filter) fiber optic probe with a ~95 ± 3% average transmittance (*T*_avg_). An Orphir Optronics Ltd (Jerusalem, Israel) Orion power meter was used to determine the fluence rate of the LC122. At a minimum, experiments were performed in triplicate, and their statistical significance was determined using an unpaired Student’s two-tailed *t*-test.

#### 3.6.1. Bacteria

Sterile, flat-bottom 24-well plates (BD Falcon) were prepared with PAN-Por^(+)^ cut to precisely fit the well bottom (∼1 cm diam.) using a custom holepunch. Aliquots (100 µL) of cell culture were transferred to each well and subjected to visible (400–700 nm, 65 ± 5 mW/cm^2^ fluence rate) light illumination for 5–60 min. Studies were repeated as described with PAN (PS-free material control), and in the absence of material (cells-only control), under both illuminated and non-illuminated (dark) conditions. After the illumination period, each well was serially diluted 1:10 five times. From each dilution, 10 µL aliquots, from the undiluted well and from the dark control, were all separately plated and incubated at 37 °C in the dark. Square agar plates (gridded six column), prepared without antibiotics and utilizing the appropriate growth media, were employed for each bacterium. The survival rate was calculated by dividing the CFU/mL of the illuminated solution by the CFU/mL of the dark control. As the plating technique employed a 10 µL aliquot from the 1 mL undiluted well, the minimum detection limit was determined to be 100 CFU/mL. Owing to variations in the starter culture concentrations spanning 1–4 × 10^8^ CFU/mL, a corresponding variation of the detection limit resulted from 0.001 to 0.0001%. Illuminated samples of PAN without the photosensitizer served as the light control, whereas samples with PAN-Por^(+)^ that were kept in the dark served as the dark control.

#### 3.6.2. Vesicular stomatitis virus

There were 25 μL of a VSV stock (5 × 10^8^ plaque forming units (PFU)/mL) added to either empty well (control), PAN, or PAN-Por^(+)^ in wells of a 96-well plate in the dark. The plates were subjected to visible (400–700 nm) light illumination for 30 min with a fluence of 65 ± 5 mW/cm^2^ for the aPDI assay, or were kept in the dark for the control experiments. Treatments were performed in biological triplicates. After illumination, 100 μL of minimum essential medium (MEM) supplemented with 1% FBS, 10 mM HEPES and antibiotics were added to wash remaining viruses off the textiles. Viruses were subsequently titered by serial dilution (10-fold) using Vero cells in 12-well plates at 37 °C. VSV concentration (detection limit of 40 PFU/mL) was determined by plaque assay. Crystal violet staining was employed to visualize the plaques 48 h after infection. For titer determination where VSV was detected, the diluted wells that contained between 10 and 30 plaques were counted.

#### 3.6.3. Human adenovirus-5

There were 25 μL of a human adenovirus 5 stock (4.5 × 10^7^ PFU/mL) added to either empty well (control), PAN or PAN-Por^(+)^ in wells of a 96 well plate in the dark. The plates were subjected to visible (400–700 nm) light illumination for 30 min with a fluence of 65 ± 5 mW/cm^2^ for the aPDI assay, or were kept in the dark for the control experiments. Treatments were performed in biological triplicates. After illumination, 100 μL of DMEM supplemented with 10% FBS and antibiotics were added to wash remaining viruses off the textiles. Viruses were subsequently titered by serial dilution (10-fold) using A549 cells in 12-well plates at 37 °C. Plaques were detected by crystal violet staining 120 h after infection. For titer determination where HAd-5 was detected, the diluted wells that contained between 10–30 plaques were counted.

## 4. Conclusions

The antimicrobial efficacy of PAN-Por^(+)^ was explored against taxonomically diverse bacteria, focusing on *E. coli* and the ESKAPE pathogens *S. aureus*, *E. faecium*, *A. baumannii*, and *K. pneumoniae*, as well as against viruses, including the non-enveloped human adenovirus-5 and the enveloped vesicular stomatitis virus. Our results demonstrated that PAN-Por^(+)^ possesses broad antibacterial efficacy, with equivalent levels of inactivation being observed regardless of the bacterial strain tested, and at levels that exceeded Por^(+)^-paper or Por^(+)^-CNCs, the two cellulose-based materials employing the same photosensitizer via a covalent linkage to the polymer scaffold. PAN-Por^(+)^ also exhibited improved antiviral efficacy compared to the cellulose-based analogs when studied against human adenovirus-5 and vesicular stomatitis virus. These findings for PAN-Por^(+)^ suggest that the combined effects of a higher photosensitizer loading achievable from a non-covalent attachment strategy of the photosensitizer, coupled with a higher surface area from using nanofibers, together enable improved antimicrobial efficacy, likely from a higher amount of singlet oxygen produced per unit area, and leads us to conclude that future iterations of these photosensitizer-embedded materials should be designed with the goal of maximizing both surface area and photosensitizer loading. Although photobleaching was minimal over the duration tested, future studies aimed at prolonging the lifespan of materials operating by a photodynamic mode of action should be explored. Taken together, the combined antimicrobial results obtained for PAN-Por^(+)^ demonstrate the promise and potential of scalable nanofiber-based aPDI materials as a platform technology for anti-infective materials: synthetic polyacrylonitrile nanofibers embedded with a cationic porphyrin photosensitizer exhibit superior antibacterial and antiviral efficacy compared to natural fiber materials, are less synthetically challenging to prepare than covalent strategies, are more easily scalable, and therefore have potential for incorporation into consumer staples that are broadly anti-infective. Future iterations and planned studies on next-generation materials will be focused on utilizing more cost-effective and commercially available photosensitizers that are capable of the rapid, efficient, and low-cost autonomous sterilization of a range of infective agents for the prevention of nosocomial infections in healthcare settings.

## Supplementary Materials

The following are available online at http://www.mdpi.com/2079-4991/6/4/77/s1.
