# Supplementary material for: Photosensitizer-Embedded Polyacrylonitrile Nanofibers as Antimicrobial Non-Woven Textile
Source: Nanomaterials (Basel). 2016 Apr 20;6(4):77. doi: 10.3390/nano6040077 (PMC5302559; doi:10.3390/nano6040077)
Supplement: Supplementary file 1 [file nanomaterials-06-00077-s001.pdf]

Supplementary Materials: Photosensitizer-Embedded Polyacrylonitrile Nanofibers as an Antimicrobial Non-Woven Textile

Sarah L. Stanley, Frank Scholle, Jiadeng Zhu, Yao Lu, Xiangwu Zhang, Xingci Situ and Reza A. Ghiladi

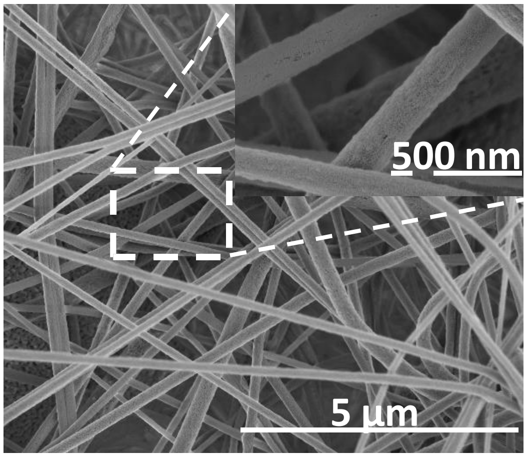

Figure S1. Scanning electron microscopy (SEM) images of electrospun polyacrylonitrile nanofibers (PAN) employed as the photosensitizer-free control material in this study. The insert represents a high resolution image.

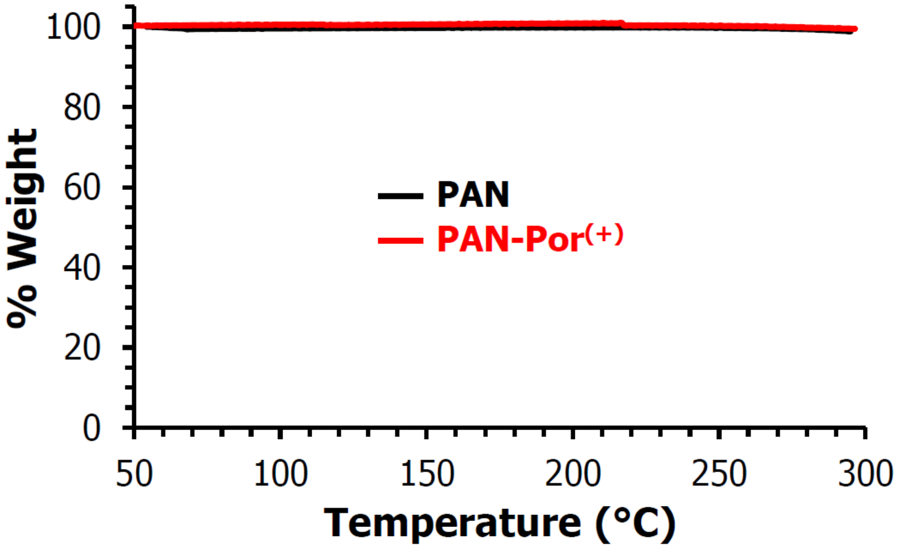

Figure S2. Thermal gravimetric analysis of PAN mother fibers (black) and PAN-Por$^{(+)}$ (red).

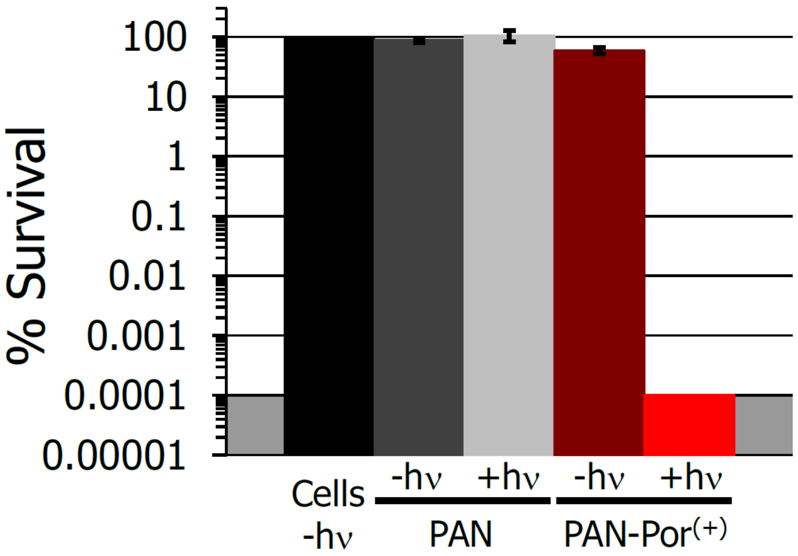

Figure S3. Photodynamic inactivation control study of *Klebsiella pneumoniae* employing both PAN and PAN-Por(+). Displayed are the material-free (cells-only) dark control set to 100% (black), as well as the dark control of PAN (dark grey), the illuminated control of PAN (light grey), the dark control of PAN-Por(+) (maroon), and the illuminated PAN-Por(+) (red), all displayed as a percent survival of the material-free dark control. Studies with error bars were performed in duplicate, and the illumination conditions were as follows: 30 min, 400–700 nm, 65 ± 5 mW/cm² (total fluence of 118 J/cm²). As the plating technique employed to determine % survival did not allow for detection of survival rates of <0.0001%, data points below the detection limit were set to 0.0001% survival for graphing purposes, corresponding to the shaded area.

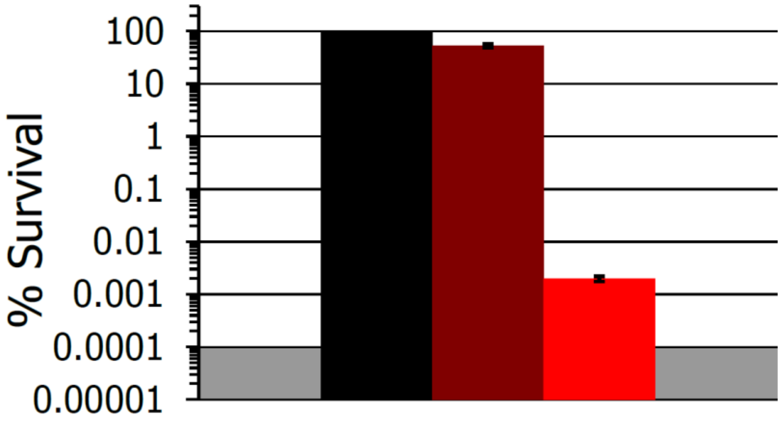

Figure S4. Photodynamic inactivation study of *Klebsiella pneumoniae* employing ‘photo-aged’ PAN-Por(+) that was pre-illuminated (400–700 nm, 65 ± 5 mW/cm²) for 8 h. Displayed are the material-free (cells-only) dark control set to 100% (black), as well as the dark control of PAN-Por(+) (maroon) and the illuminated photo-aged PAN-Por(+) (red) studies, both as a percent of the material-free (cells-only) dark control. For all studies, the illumination conditions were as follows: 30 min, 400–700 nm, 65 ± 5 mW/cm² (total fluence of 118 J/cm²). As the plating technique employed to determine % survival did not allow for detection of survival rates of <0.0001%, data points below the detection limit were set to 0.0001% survival for graphing purposes, corresponding to the shaded area.
